# Spatiotemporal patterns and future trends of global ischemic stroke burden

**DOI:** 10.3389/fneur.2026.1800093

**Published:** 2026-04-20

**Authors:** Ruojing Zhang, Wenjun Chen, Mingjin Zhu, Yufei Xu

**Affiliations:** 1 Department of Rehabilitation Medicine, Tongde Hospital of Zhejiang Province, Hangzhou, China; 2 Department of Pharmacy, Xixi Hospital of Hangzhou, Hangzhou, China

**Keywords:** epidemiological trends, gender and age disparities, global burden, ischemic stroke, sociodemographic index

## Abstract

**Background:**

Ischemic stroke remains a leading cause of morbidity and mortality worldwide, with substantial heterogeneity across regions, sexes, age groups, and levels of socioeconomic development. Previous studies have described global trends; however, integrated analyses combining long-term spatiotemporal patterns, sociodemographic stratification, and future projections remain limited.

**Methods:**

Data on ischemic stroke from 1990 to 2021 were extracted from the Global Burden of Disease (GBD) 2021 database, covering 204 countries and territories. Age-standardized prevalence, incidence, mortality, and disability-adjusted life years (DALYs) were analyzed by age, sex, region, and sociodemographic index (SDI). Temporal trends were assessed using estimated annual percentage change (EAPC). Future disease burden from 2022 to 2041 was projected using autoregressive integrated moving average (ARIMA) models implemented with the R package forecast (version 8.24.0).

**Results:**

Globally, from 1990 to 2021, age-standardized incidence declined from 109.79 to 92.39 per 100,000 (EAPC = −0.67), mortality from 73.15 to 44.18 per 100,000 (EAPC = −1.83), and DALYs from 1286.31 to 837.36 per 100,000 (EAPC = −1.59), whereas prevalence showed only a modest decrease (EAPC = −0.18). Marked disparities were observed across SDI levels: middle SDI regions experienced increasing prevalence (EAPC = 0.27) and incidence (EAPC = 0.12), while high SDI regions demonstrated the most pronounced reductions in mortality and DALYs. Eastern Europe, East Asia, and Southern Sub-Saharan Africa remained high-burden regions. Pronounced sex- and age-specific patterns were identified, with men exhibiting earlier peak incidence (65–79 years) and women showing later and shifting peak prevalence toward older age groups. Projections indicate that by 2041, global age-standardized prevalence will increase by approximately 15.3%, driven primarily by a substantial rise among females (from 769.40 to 1005.77 per 100,000), despite continued declines in mortality and DALYs.

**Conclusion:**

This comprehensive spatiotemporal analysis reveals a paradoxical pattern of increasing ischemic stroke prevalence amid declining mortality and disability, with widening sex- and SDI-related disparities. By integrating long-term trends with sex-specific future projections, this study provides novel evidence to inform targeted prevention strategies, gender-sensitive interventions, and context-specific health policy planning to mitigate the evolving global burden of ischemic stroke.

## 1 Introduction

Ischemic stroke is a leading cause of death and disability worldwide, imposing a substantial and persistent global public health burden. In 2021, approximately 69.9 million people were living with ischemic stroke, corresponding to an age-standardized prevalence rate of 819.5 per 100,000 population (1). The age-standardized incidence rate was 92.4 per 100,000, with a mortality rate of 44.2 per 100,000 and an age-standardized disability-adjusted life years (DALYs) rate of 837.4 per 100,000 (1). Although there has been a declining trend in age-standardized rates since 1990—reflected in an estimated annual percentage change of −0.67% for incidence and −1.83% for mortality (2)—the absolute number of ischemic stroke cases has increased by 47.14% over recent decades (3), largely due to population growth and aging. The burden of ischemic stroke is distributed unevenly across regions and socioeconomic strata: high-middle sociodemographic index (SDI) regions report the highest disease rates, while high SDI regions have the lowest (4). Certain areas, such as Eastern Europe, Central Asia, and parts of Sub-Saharan Africa, bear especially high burdens (2). Marked gender disparities are observed, with men experiencing higher incidence and DALYs rates than women, and the male-to-female ASDR ratio increasing from 1.10 in 1990 to 1.21 in 2019 (5). Major contributing risk factors include high systolic blood pressure, elevated LDL cholesterol, smoking, and, in lower SDI regions, household air pollution (4). The clear inverse correlation between ischemic stroke burden and economic development emphasizes the critical impact of socioeconomic factors on prevention, diagnosis, and access to care (2). These patterns are further intensified by ongoing global trends such as population aging and changing lifestyles, highlighting the urgency of in-depth investigation into the evolving burden of ischemic stroke.

Despite notable advances in understanding the epidemiology of ischemic stroke, important knowledge gaps and limitations persist in the current literature. Prior studies have documented the global burden and regional differences of ischemic stroke (1), yet few have provided comprehensive, up-to-date analyses across diverse socioeconomic contexts. Recent work highlights significant geographic disparities, with high-middle SDI regions experiencing the greatest age-standardized prevalence, incidence, mortality, and DALY rates (1); however, many analyses fail to capture the complex interplay between socioeconomic factors and stroke outcomes. Temporal trends have often been examined without sufficient attention to variations across age and gender groups (6). For instance, the rising incidence among young adults (15–39 years) has been underrepresented in research (6). Moreover, while some studies have projected future trends, these projections typically lack stratification by key demographic and socioeconomic variables (7). Methodological inconsistencies and heterogeneous data sources have further limited robust cross-study comparisons (8). The impact of emerging risk factors and the COVID-19 pandemic on ischemic stroke epidemiology has also not been fully addressed, with preliminary evidence suggesting a potential increase in stroke burden during the pandemic period (9). Additionally, country-specific analyses remain limited, particularly in regions undergoing rapid socioeconomic transitions (10). Collectively, these limitations underscore the need for updated, comprehensive analyses that address temporal trends, demographic and geographic disparities, and future projections of ischemic stroke burden in various socioeconomic settings.

The sociodemographic index (SDI) serves as a vital composite measure of regional socioeconomic development and is closely linked to disparities in ischemic stroke burden worldwide. Integrating income per capita, educational attainment, and fertility rates, SDI allows for nuanced assessment of how social and economic factors shape health outcomes (8). The relationship between SDI and ischemic stroke burden is complex: a reversed V-shaped association has been observed, with middle-SDI regions bearing the highest age-standardized rates, while both low and high SDI regions experience comparatively lower burdens (11). This non-linear pattern underscores the multifaceted effects of socioeconomic transitions on disease risk and outcomes. Socioeconomic factors influence the prevention, diagnosis, and management of ischemic stroke through multiple pathways. In lower-SDI regions, limited healthcare infrastructure and reduced access to specialized care can delay diagnosis and lead to suboptimal treatment (12), while higher-SDI regions benefit from advanced medical technologies and established care protocols, resulting in improved outcomes (13). The prevalence and impact of risk factors, such as hypertension and household air pollution, also vary across SDI categories (14). Despite global declines in age-standardized rates, significant disparities persist between regions at different stages of socioeconomic development (11). Understanding these disparities is crucial for developing targeted interventions and allocating resources effectively to reduce the global burden of ischemic stroke.

In this context, the present study aims to provide a comprehensive and updated assessment of the global burden of ischemic stroke using GBD 2021 data from 1990 to 2021, with projections extending to 2041. This study offers several advances beyond prior GBD-based analyses. First, we identify a critical paradox: despite declining mortality and DALYs, global age-standardized prevalence is projected to increase substantially through 2041—a divergence with major implications for health systems. Second, we provide the first sex-specific forecasts to 2041, revealing a striking surge in female prevalence that has not been previously quantified. Third, our integrated SDI-stratified analysis pinpoints middle-SDI regions at an epidemiological inflection point, where rising incidence coincides with limited preventive infrastructure. Fourth, we systematically characterize shifting age peaks by sex and SDI, identifying a progressive delay in women’s peak prevalence that reflects the convergence of biological, reproductive, and healthcare factors. These findings collectively move beyond describing disparities to identifying specific populations and regions at critical intervention windows.

## 2 Methods

### 2.1 Data collection

To investigate the impact of ischemic stroke, we utilized data from the Global Burden of Disease (GBD) database spanning 1990 to 2021.^1^ Since 1990, the GBD study has continuously provided annual estimates for multiple disease metrics, offering a comprehensive and comparable assessment of disease burden and trends at global, regional, and national levels. We employed data-driven indicators for ischemic stroke, including age-standardized prevalence rate (ASPR), age-standardized incidence rate (ASIR), age-standardized death rate (ASDR), and disability-adjusted life years (DALYs) per 100,000 population. This analysis covered 204 countries and territories, categorized into 21 GBD regions based on geographic proximity and further classified into five categories according to the sociodemographic index (SDI).

### 2.2 Sociodemographic index

The SDI is a composite measure developed by the GBD research team to assess the socioeconomic status of regions. It integrates income per capita, educational attainment, and fertility rate into a single value ranging from 0 to 1, reflecting the socioeconomic development and health status of a region or country (15, 16). Higher SDI values indicate superior socioeconomic conditions and health outcomes. SDI divides regions into five quintiles: low SDI (0–0.454743), low-middle SDI (0.454743–0.607679), middle SDI (0.607679–0.689504), high-middle SDI (0.689504–0.805129), and high SDI (0.805129–1).

### 2.3 Statistical analysis

#### 2.3.1 Trend analysis with estimated annual percentage change

To evaluate temporal trends in the age-standardized rates (ASR) of ischemic stroke prevalence, incidence, mortality, and DALYs, we applied the estimated annual percentage change (EAPC) method (17). The regression model used was: *Y* = *α* + *β*X + *e*, where *Y* represents the natural logarithm of ASR, *X* is the calendar year, *α* is the intercept, *β* is the slope (trend), and e is the error term. EAPC was calculated as 100 × [exp(*β*) – 1], indicating the annual percentage change. Linear regression was utilized to estimate the 95% confidence interval (CI) of EAPC. An upward trend in ASR was defined when both EAPC and the lower limit of the 95% CI were positive; a downward trend was defined when both EAPC and the upper limit of the 95% CI were negative; otherwise, the ASR was considered stable.

#### 2.3.2 Descriptive analysis of disease variation

To preliminarily characterize the epidemiology of ischemic stroke, we conducted descriptive analyses of total case counts and age-specific case numbers reported since 1990. Percentage change during the study period was calculated using the formula: percentage change = (value in current year − value in previous year)/value in previous year × 100%. Age groups were categorized in 5-year intervals, totaling 20 groups ranging from <5 years to ≥95 years.

#### 2.3.3 Forecasting future disease burden

We employed the R package forecast (version 8.24.0) to predict the prevalence, incidence, mortality, and DALYs of ischemic stroke over the next 20 years (2022–2041) using autoregressive integrated moving average (ARIMA) models. Prior to modeling, we assessed the stationarity of each time series (global age-standardized rates for prevalence, incidence, mortality, and DALYs) using the augmented Dickey–Fuller test. No logarithmic or other variance-stabilizing transformations were applied to the raw age-standardized rate (ASR) data in order to maintain interpretability of the original units. For objective and replicable model selection, we utilized the auto.arima() function. This algorithm automatically identifies the optimal ARIMA parameters (autoregressive order, p; differencing order, d; moving average order, q) by fitting a range of candidate models and selecting the one that minimizes the corrected Akaike information criterion (AICc). The function handles the selection of the order of differencing (d) to achieve stationarity. The final selected models were validated by checking the residuals for white noise using the Ljung–Box test. Forecasts were generated with 95% confidence intervals to represent uncertainty, and trend plots were constructed with the *X*-axis representing years from 1990 to 2041 (displaying both historical data and projections) and the *Y*-axis representing the values of the selected indicators. This forecasting approach aids in better understanding potential trends and provides a reference for the development of related interventions.

## 3 Results

### 3.1 Global trends from 1990 to 2021

**Figure 1:**
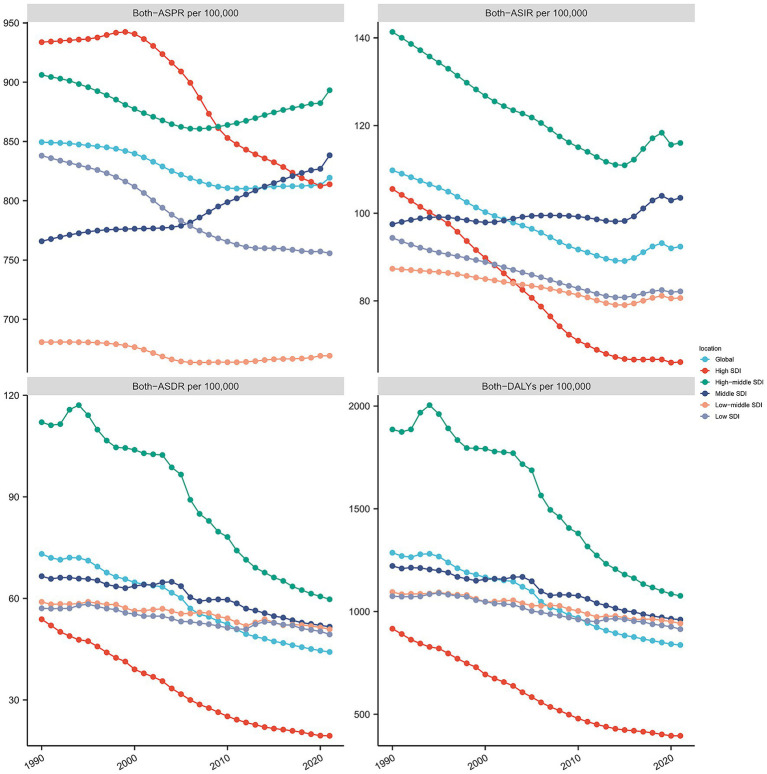
Trends in ischemic stroke prevalence, incidence, mortality, and disability-adjusted life years (DALYs) from 1990 to 2021.

From 1990 to 2021, the global age-standardized prevalence rate (ASPR) of ischemic stroke showed a slight declining trend, decreasing from 849.49 to 819.47 per 100,000 population, with an estimated annual percentage change (EAPC) of −0.1834, indicating a modest reduction in prevalence. During the same period, the age-standardized incidence rate (ASIR) declined more markedly from 109.79 to 92.39 per 100,000, with an EAPC of −0.6735, reflecting a more pronounced decrease in incidence. The reductions in age-standardized death rate (ASDR) and age-standardized disability-adjusted life years (DALYs) rate were even more substantial, falling from 73.15 to 44.18 per 100,000 (EAPC = −1.8283) and from 1286.31 to 837.36 per 100,000 (EAPC = −1.5913), respectively, suggesting a significant alleviation of mortality and disease burden attributable to ischemic stroke (Table 1 and Figure 1).

**Table 1:** Global trends in prevalence, incidence, mortality, and disability-adjusted life years (DALYs) of ischemic stroke burden in 1990 and 2021.

Measure	Sex	1990 number (95% UI)	1990 ASR (95% UI)	2021 number (95% UI)	2021 ASR (95% UI)	EAPC (95% CI)
Prevalence	Male	16806787.39 (15523886.04, 18032951.70)	905.01 (833.61, 973.14)	35241152.37 (32609808.78, 37813501.27)	881.94 (818.60, 944.54)	−0.1164 (−0.1304, −0.1023)
Prevalence	Female	17861253.98 (16549761.13, 19146767.48)	812.28 (754.34, 870.75)	34703732.45 (32096979.39, 37274015.32)	769.40 (712.98, 825.99)	−0.2771 (−0.3273, −0.2270)
Prevalence	Both	34668041.37 (32153636.82, 37171587.51)	849.49 (785.92, 913.25)	69944884.82 (64788695.13, 75009602.78)	819.47 (760.26, 878.71)	−0.1834 (−0.2126, −0.1541)
Incidence	Male	2011054.18 (1696281.56, 2365175.63)	117.02 (99.30, 136.42)	4022421.46 (3444603.46, 4666933.17)	102.77 (88.65, 118.80)	−0.4820 (−0.5386, −0.4255)
Incidence	Female	2140923.90 (1826404.07, 2500901.43)	102.87 (87.94, 119.63)	3782027.95 (3253626.36, 4336192.77)	82.85 (71.31, 94.81)	−0.8723 (−1.0051, −0.7393)
Incidence	Both	4151978.08 (3536772.36, 4868149.60)	109.79 (93.56, 127.62)	7804449.40 (6719760.38, 8943692.09)	92.39 (79.84, 105.82)	−0.6735 (−0.7664, −0.5804)
Deaths	Male	1007453.27 (922680.55, 1107726.86)	76.50 (70.09, 83.14)	1778734.98 (1611776.98, 1962926.18)	51.16 (46.21, 56.22)	−1.4437 (−1.5231, −1.3641)
Deaths	Female	1309659.01 (1194495.13, 1394755.89)	69.89 (62.93, 74.40)	1812763.64 (1573025.20, 2001279.29)	38.54 (33.46, 42.53)	−2.1644 (−2.2770, −2.0516)
Deaths	Both	2317112.28 (2131459.55, 2475545.55)	73.15 (66.36, 77.94)	3591498.62 (3213281.25, 3888326.82)	44.18 (39.29, 47.81)	−1.8283 (−1.9236, −1.7329)
DALYs	Male	21880293.41 (20063438.24, 24119195.05)	1383.23 (1267.48, 1510.01)	37007430.04 (33581055.23, 40659722.80)	975.30 (885.61, 1069.81)	−1.2840 (−1.3640, −1.2040)
DALYs	Female	24295946.83 (22551181.72, 26052676.31)	1199.95 (1105.61, 1284.13)	33350481.84 (29775875.10, 36653245.30)	719.52 (642.82, 791.64)	−1.9031 (−2.0102, −1.7959)
DALYs	Both	46176240.24 (42961948.38, 49414586.11)	1286.31 (1195.19, 1376.06)	70357911.88 (64329575.55, 76007063.01)	837.36 (763.73, 904.98)	−1.5913 (−1.6826, −1.5000)

ASR, age-standardized rate (per 100,000 person-years); EAPC, estimated annual percentage change; UI, uncertainty interval; CI, confidence interval; DALYs, disability-adjusted life years.

Source: Global Burden of Disease Study 2021.

EAPC indicates the average annual percentage change in ASR from 1990 to 2021.

Ischemic stroke burden and its temporal trends vary significantly across SDI-level regions, with middle SDI areas showing increasing disease burden, whereas high SDI regions demonstrate declines. These differences likely reflect disparities in healthcare resource allocation, preventive measures, and lifestyle changes. The concentration of burden in specific regions such as Sub-Saharan Africa and Eastern Europe points to critical public health challenges requiring focused prevention and treatment efforts.

These data reflect continuous global declines in incidence, prevalence, mortality, and DALYs of ischemic stroke, likely attributable to improvements in public health interventions, healthcare quality, and risk factor control. Despite overall favorable trends, the pace of decline varies across different regions and SDI levels, indicating the need for tailored preventive and control strategies to further reduce the ischemic stroke burden.

### 3.2 Regional trends

#### 3.2.1 Burden stratified by SDI quintiles

Data from the GBD database reveal marked regional disparities in the global burden of ischemic stroke closely linked to SDI levels. Among SDI categories, the highest ASPR was observed in high-middle SDI regions at 893.25 (range: 824.01–959.98) per 100,000, whereas the lowest was in low-middle SDI regions at 669.30 (616.25–720.63) per 100,000. Notably, middle SDI regions exhibited the most rapid increase in ASPR from 1990 to 2021 [estimated annual percentage change (EAPC) = 0.27].

Similar patterns were observed for incidence, mortality, and DALYs. High-middle SDI regions consistently bore the heaviest burden, with the highest incidence (116.04 per 100,000), mortality (59.75 per 100,000), and DALY rates (1076.54 per 100,000) in 2021. Conversely, high SDI regions recorded the lowest rates across all metrics (incidence: 66.05; mortality: 19.42; DALYs: 395.56 per 100,000) and demonstrated the steepest declines over the study period (EAPC for mortality = −3.58; EAPC for DALYs = −2.98). In contrast, middle SDI regions showed an increasing trend in incidence (EAPC = 0.12). For complete data on all SDI quintiles, see Table 2.

**Table 2:** Regional trends in prevalence, incidence, mortality, and disability-adjusted life years (DALYs) of ischemic stroke burden in 1990 and 2021.

Measure	Location	1990 number (95% UI)	1990 ASR (95% UI)	2021 number (95% UI)	2021 ASR (95% UI)	EAPC (95% CI)
Prevalence	Central Asia	550460.48 (523473.42, 577216.79)	1107.87 (1052.26, 1164.94)	866712.43 (829519.17, 902763.03)	1014.86 (969.90, 1063.57)	−0.3124 (−0.3323 to −0.2925)
Prevalence	High-income Asia Pacific	2058456.09 (1893145.48, 2220222.19)	1030.51 (947.08, 1109.23)	3295961.70 (3048492.74, 3555085.79)	775.63 (722.99, 832.15)	−1.0376 (−1.0949 to −0.9803)
Prevalence	Southeast Asia	2557635.51 (2350464.33, 2762167.00)	921.18 (846.81, 999.59)	6104965.95 (5651318.63, 6562856.99)	919.03 (851.92, 986.33)	−0.0288 (−0.0371 to −0.0206)
Prevalence	Central Latin America	678691.88 (630061.57, 725121.03)	725.24 (673.94, 777.84)	1387551.69 (1296756.25, 1484316.02)	552.56 (516.91, 591.30)	−1.0268 (−1.0926 to −0.9608)
Prevalence	Central Europe	1418231.69 (1324818.37, 1513619.57)	959.62 (897.79, 1022.43)	1631106.09 (1537835.00, 1733826.69)	776.07 (733.54, 820.85)	−0.7709 (−0.8227 to −0.7191)
Prevalence	Western Sub-Saharan Africa	1111030.51 (1034246.31, 1188452.98)	1109.41 (1026.89, 1194.52)	2479408.31 (2331910.99, 2621732.85)	1045.98 (977.73, 1112.64)	−0.2259 (−0.2396 to −0.2122)
Prevalence	High-income North America	3403854.63 (3107281.67, 3724886.19)	991.52 (904.49, 1081.88)	5699097.65 (5260863.25, 6152298.26)	949.99 (880.58, 1024.50)	−0.3917 (−0.5765 to −0.2064)
Prevalence	Southern Sub-Saharan Africa	364210.13 (330935.45, 399540.45)	1274.58 (1149.82, 1404.06)	640169.67 (583548.49, 694855.40)	1121.97 (1019.94, 1224.88)	−0.489 (−0.5376 to −0.4404)
Prevalence	Tropical Latin America	827663.72 (739294.21, 917728.81)	850.01 (757.77, 943.57)	1543559.50 (1392394.60, 1692607.61)	605.71 (546.63, 663.92)	−1.2397 (−1.3035 to −1.1758)
Prevalence	Eastern Sub-Saharan Africa	867800.77 (811693.63, 925063.38)	1066.61 (996.15, 1139.76)	1869299.31 (1761941.32, 1973402.30)	992.11 (934.22, 1049.93)	−0.2696 (−0.2922 to −0.2469)
Prevalence	Central Sub-Saharan Africa	266923.73 (253028.19, 281576.47)	1113.57 (1052.23, 1178.44)	596223.32 (568897.82, 624228.70)	995.72 (947.71, 1046.32)	−0.4062 (−0.4338 to −0.3786)
Prevalence	Oceania	26485.14 (25138.30, 27927.27)	809.68 (767.68, 854.97)	59506.26 (57089.06, 62156.10)	741.44 (711.42, 771.98)	−0.3084 (−0.3151 to −0.3018)
Prevalence	Australasia	176175.40 (169750.39, 183601.46)	751.35 (724.29, 783.41)	296950.74 (286363.03, 308252.27)	581.91 (560.18, 603.18)	−0.9349 (−0.9847 to −0.8851)
Prevalence	Middle SDI	8555789.75 (7763206.52, 9309792.16)	765.88 (693.01, 837.51)	22230670.66 (20239639.37, 24119856.05)	838.38 (764.78, 911.01)	0.2715 (0.2403 to 0.3028)
Prevalence	Andean Latin America	138242.81 (131026.60, 145937.07)	604.93 (573.40, 638.13)	312484.91 (297506.23, 327199.82)	515.11 (490.59, 538.97)	−0.6013 (−0.6387 to −0.5638)
Prevalence	Caribbean	178810.03 (169859.67, 188128.68)	649.08 (616.10, 684.12)	327164.53 (312540.55, 341547.59)	616.95 (589.70, 643.62)	−0.1813 (−0.2031 to −0.1594)
Prevalence	Eastern Europe	2869099.36 (2565924.59, 3174157.03)	1028.01 (921.85, 1132.83)	3049360.45 (2763322.37, 3340261.45)	920.45 (835.80, 1005.34)	−0.3779 (−0.4096 to −0.3463)
Prevalence	South Asia	3441862.98 (3041705.01, 3832962.84)	526.01 (462.25, 586.94)	7828397.31 (6997064.50, 8621267.46)	497.74 (445.71, 549.03)	−0.212 (−0.2497 to −0.1743)
Prevalence	North Africa and Middle East	1847829.45 (1741882.35, 1963338.43)	909.31 (852.40, 967.58)	4366507.27 (4157817.77, 4571157.71)	866.30 (823.67, 909.40)	−0.1841 (−0.2 to −0.1683)
Prevalence	Southern Latin America	399118.02 (380518.40, 419432.08)	859.38 (819.62, 903.12)	546568.68 (524188.77, 570536.20)	640.57 (614.79, 668.14)	−1.0506 (−1.1053 to −0.9958)
Prevalence	Low SDI	2124875.91 (1976569.52, 2270871.05)	838.06 (776.26, 901.21)	4447699.19 (4174214.72, 4699709.45)	755.73 (707.71, 800.06)	−0.394 (−0.4297 to −0.3582)
Prevalence	East Asia	6921707.68 (6208387.01, 7617523.78)	774.23 (691.49, 863.72)	21503822.78 (19292836.48, 23714701.29)	1017.96 (919.97, 1120.07)	0.9479 (0.8921 to 1.0037)
Prevalence	Low-middle SDI	4631395.15 (4229093.54, 5023596.59)	680.79 (620.57, 741.66)	10234283.14 (9418964.27, 11004022.09)	669.30 (616.25, 720.63)	−0.0881 (−0.1125 to −0.0637)
Prevalence	High-middle SDI	9174384.97 (8429846.23, 9882568.05)	906.21 (834.66, 975.02)	17110640.32 (15738888.30, 18478803.40)	893.25 (824.01, 959.98)	−0.0904 (−0.1451 to −0.0356)
Prevalence	High SDI	10141955.65 (9487690.00, 10833007.61)	933.82 (874.48, 998.01)	15864865.47 (14925797.92, 16872854.66)	813.89 (768.62, 864.10)	−0.5696 (−0.6409 to −0.4983)
Prevalence	Western Europe	4563751.35 (4343952.82, 4819225.20)	806.89 (768.41, 850.59)	5540066.28 (5333481.66, 5752361.87)	625.74 (600.05, 649.86)	−0.8578 (−0.8877 to −0.8279)
Incidence	Central Asia	65958.56 (58343.68, 73949.48)	141.86 (124.60, 159.13)	102572.62 (91018.37, 114462.71)	132.92 (118.17, 148.10)	−0.2006 (−0.2959 to −0.1052)
Incidence	High-income Asia Pacific	234946.27 (193847.97, 280991.00)	120.98 (100.70, 143.98)	284444.72 (249282.53, 322677.76)	64.59 (56.36, 73.72)	−2.5298 (−2.7386 to −2.3207)
Incidence	Southeast Asia	265778.95 (228020.33, 310151.07)	107.32 (91.50, 125.56)	667263.02 (582280.63, 763034.94)	107.31 (93.68, 122.69)	−0.0351 (−0.0725 to 0.0023)
Incidence	Central Latin America	68642.87 (58878.79, 78850.51)	80.45 (69.05, 93.22)	127074.90 (110154.99, 144196.40)	51.96 (45.09, 58.83)	−1.6667 (−1.829 to −1.5041)
Incidence	Central Europe	222510.34 (193304.80, 251309.21)	157.98 (138.35, 177.71)	238905.23 (209922.34, 266603.54)	107.00 (95.27, 118.40)	−1.3527 (−1.4056 to −1.2997)
Incidence	Western Sub-Saharan Africa	98569.94 (83977.20, 114606.64)	104.07 (88.24, 122.05)	208264.82 (181390.62, 236453.63)	94.74 (82.14, 106.92)	−0.2925 (−0.3319 to −0.2531)
Incidence	High-income North America	315394.24 (259208.02, 383545.11)	89.95 (74.41, 108.35)	352963.34 (299100.01, 413294.44)	56.86 (48.63, 66.11)	−1.5831 (−1.7391 to −1.4267)
Incidence	Southern Sub-Saharan Africa	33295.01 (28141.12, 39224.65)	122.29 (101.93, 146.66)	64729.44 (54456.25, 74990.50)	121.86 (102.46, 142.18)	−0.1085 (−0.3738 to 0.1575)
Incidence	Tropical Latin America	105999.96 (88155.85, 125886.08)	119.16 (98.34, 142.27)	166748.85 (140997.68, 194698.40)	66.42 (55.99, 77.52)	−2.0401 (−2.1624 to −1.9177)
Incidence	Eastern Sub-Saharan Africa	85161.60 (72719.03, 99207.23)	110.88 (94.18, 130.12)	179317.52 (154965.56, 204334.22)	103.55 (89.22, 118.40)	−0.2143 (−0.2647 to −0.1639)
Incidence	Central Sub-Saharan Africa	27144.27 (23222.02, 32004.74)	122.67 (104.25, 142.58)	59172.89 (51496.17, 68041.34)	108.99 (94.95, 124.84)	−0.4147 (−0.4482 to −0.3813)
Incidence	Oceania	2342.92 (2016.86, 2728.23)	81.50 (70.05, 94.27)	5311.49 (4624.94, 6003.23)	73.20 (63.72, 82.91)	−0.4329 (−0.4732 to −0.3926)
Incidence	Australasia	20989.30 (19362.09, 22541.04)	91.09 (84.12, 97.82)	28159.56 (25187.70, 31108.96)	52.76 (47.35, 58.52)	−2.0519 (−2.2043 to −1.8992)
Incidence	Middle SDI	953540.58 (800911.74, 1134826.89)	97.51 (81.45, 115.65)	2648524.64 (2240569.11, 3082586.97)	103.51 (88.01, 119.98)	0.1168 (0.0673 to 0.1664)
Incidence	Andean Latin America	13562.92 (11785.17, 15387.02)	63.46 (55.42, 71.89)	27415.48 (24041.95, 31116.17)	46.29 (40.60, 52.41)	−1.1292 (−1.2413 to −1.0169)
Incidence	Caribbean	19577.40 (17215.68, 21952.58)	76.19 (67.54, 85.71)	36042.25 (32142.46, 39960.60)	67.34 (59.96, 74.74)	−0.4189 (−0.466 to −0.3718)
Incidence	Eastern Europe	515846.23 (425258.35, 623600.77)	197.90 (164.89, 232.39)	490197.06 (415356.09, 571450.55)	142.57 (122.12, 164.67)	−1.134 (−1.248 to −1.0199)
Incidence	South Asia	398076.36 (331671.95, 474038.32)	72.63 (60.60, 86.45)	853370.32 (727638.35, 990208.94)	61.16 (52.33, 70.75)	−0.7413 (−0.8241 to −0.6583)
Incidence	North Africa and Middle East	199912.22 (175527.71, 229003.37)	117.53 (102.61, 133.52)	461145.62 (409815.06, 516836.61)	103.58 (91.57, 116.06)	−0.4335 (−0.4765 to −0.3906)
Incidence	Southern Latin America	44172.32 (38720.00, 49910.24)	97.84 (86.31, 109.85)	52069.93 (46168.93, 58185.00)	60.35 (53.46, 67.61)	−1.774 (−1.9322 to −1.6156)
Incidence	Low SDI	216493.47 (183974.30, 252019.00)	94.39 (80.38, 110.81)	433298.39 (374564.81, 494218.02)	82.17 (71.04, 93.10)	−0.5115 (−0.5642 to −0.4588)
Incidence	East Asia	800330.32 (656281.05, 982072.71)	101.31 (82.99, 121.88)	2850089.70 (2363157.65, 3405882.49)	134.77 (112.62, 158.40)	0.8614 (0.8004 to 0.9225)
Incidence	Low-middle SDI	516850.86 (439585.90, 603885.19)	87.34 (74.13, 102.39)	1120427.19 (971629.86, 1273722.47)	80.66 (69.98, 91.67)	−0.3384 (−0.3792 to −0.2975)
Incidence	High-middle SDI	1306276.52 (1096265.30, 1547833.19)	141.37 (119.89, 164.14)	2243339.86 (1899825.15, 2614462.65)	116.04 (99.23, 133.86)	−0.7403 (−0.852 to −0.6286)
Incidence	High SDI	1153374.19 (992805.96, 1333283.40)	105.54 (91.16, 121.17)	1351912.50 (1179760.97, 1535709.83)	66.05 (58.24, 74.72)	−1.7505 (−1.869 to −1.632)
Incidence	Western Europe	613766.10 (538423.49, 692797.70)	105.97 (93.99, 117.98)	549190.63 (496801.87, 601605.62)	58.14 (52.88, 63.67)	−2.1243 (−2.2185 to −2.03)
Deaths	Central Asia	32985.70 (30490.77, 35153.18)	81.73 (74.95, 87.16)	44869.98 (40632.43, 48966.02)	70.96 (64.29, 77.28)	−0.871 (−1.1167 to −0.6248)
Deaths	High-income Asia Pacific	105658.85 (93890.93, 112114.08)	62.85 (54.94, 67.16)	112784.62 (87154.88, 127257.96)	15.77 (12.54, 17.52)	−4.755 (−4.9142 to −4.5955)
Deaths	Southeast Asia	131563.03 (114807.69, 147288.50)	72.59 (62.95, 81.65)	341540.97 (293259.04, 391952.88)	68.09 (58.72, 77.47)	−0.1284 (−0.2824 to 0.0259)
Deaths	Central Latin America	24285.28 (22792.67, 25098.45)	37.50 (34.90, 38.88)	43026.92 (37956.30, 47739.92)	18.68 (16.48, 20.72)	−2.3935 (−2.5678 to −2.2188)
Deaths	Central Europe	181353.16 (171926.58, 186859.24)	140.97 (132.21, 145.88)	160199.81 (144922.23, 171305.79)	65.59 (59.35, 70.12)	−2.7861 (−2.9305 to −2.6414)
Deaths	Western Sub-Saharan Africa	49286.86 (38585.33, 64037.95)	76.45 (60.17, 98.52)	93012.14 (77667.86, 111240.96)	68.24 (58.16, 80.98)	−0.3578 (−0.4525 to −0.263)
Deaths	High-income North America	110126.62 (96139.90, 117560.50)	29.57 (25.75, 31.61)	126352.93 (103455.01, 138349.40)	16.76 (13.85, 18.27)	−2.3832 (−2.6967 to −2.0687)
Deaths	Southern Sub-Saharan Africa	9795.30 (8049.85, 11101.04)	47.29 (38.49, 53.89)	24959.14 (22677.35, 27158.07)	60.31 (54.56, 65.54)	0.9436 (0.4453 to 1.4445)
Deaths	Tropical Latin America	55341.27 (50933.99, 57540.95)	80.36 (72.60, 84.33)	71582.29 (63128.33, 76434.99)	29.61 (26.03, 31.67)	−3.0218 (−3.142 to −2.9014)
Deaths	Eastern Sub-Saharan Africa	24975.26 (20418.54, 31170.83)	51.86 (42.85, 64.28)	51922.75 (43067.00, 61814.25)	46.35 (38.28, 55.34)	−0.444 (−0.4837 to −0.4044)
Deaths	Central Sub-Saharan Africa	8561.31 (6432.00, 10967.92)	64.74 (49.96, 81.82)	18858.60 (13939.73, 25744.90)	59.61 (44.16, 81.52)	−0.408 (−0.4624 to −0.3536)
Deaths	Oceania	805.67 (627.05, 1041.46)	48.66 (39.30, 62.05)	1843.00 (1487.89, 2388.65)	40.70 (33.33, 52.33)	−0.6908 (−0.7577 to −0.6239)
Deaths	Australasia	9883.41 (8879.51, 10632.66)	45.74 (40.52, 49.31)	9400.17 (7587.51, 10374.54)	14.11 (11.44, 15.55)	−4.006 (−4.1175 to −3.8944)
Deaths	Middle SDI	484058.83 (438722.61, 543562.92)	66.56 (59.51, 74.59)	1171548.25 (1037070.42, 1296280.05)	51.64 (45.40, 57.09)	−0.8563 (−0.9526 to −0.76)
Deaths	Andean Latin America	5509.88 (4929.87, 6092.15)	31.72 (28.45, 34.97)	9793.99 (8202.41, 11613.60)	17.73 (14.85, 21.01)	−2.1124 (−2.3152 to −1.9091)
Deaths	Caribbean	11986.14 (11099.08, 12742.06)	52.96 (48.88, 56.13)	19947.86 (17601.23, 22402.63)	36.42 (32.14, 40.94)	−1.1632 (−1.2407 to −1.0856)
Deaths	Eastern Europe	405262.28 (383950.04, 415283.82)	168.09 (157.84, 173.00)	329291.17 (299910.79, 356034.73)	90.99 (82.79, 98.48)	−2.7841 (−3.2615 to −2.3043)
Deaths	South Asia	172616.57 (142119.86, 218916.23)	43.32 (35.65, 54.10)	441295.82 (382997.08, 539466.95)	37.98 (33.12, 45.77)	−0.5675 (−0.6891 to −0.4458)
Deaths	North Africa and Middle East	131134.67 (114693.26, 148486.89)	106.62 (92.27, 120.78)	253284.04 (220811.75, 283440.69)	73.69 (63.97, 82.06)	−1.1718 (−1.2311 to −1.1125)
Deaths	Southern Latin America	23299.96 (21541.00, 24643.93)	57.53 (52.69, 60.88)	19658.02 (17471.49, 21165.17)	21.14 (18.83, 22.77)	−2.8739 (−3.0263 to −2.7213)
Deaths	Low SDI	87274.64 (72464.97, 110945.66)	57.07 (47.48, 72.01)	174655.23 (149332.81, 216890.55)	49.38 (42.13, 60.35)	−0.4792 (−0.5451 to −0.4132)
Deaths	East Asia	442486.27 (376804.16, 522373.69)	74.59 (64.10, 87.38)	1202218.46 (1010916.23, 1397915.17)	63.18 (52.91, 73.15)	−0.517 (−0.7727 to −0.2606)
Deaths	Low-middle SDI	258770.86 (227017.37, 300181.57)	58.98 (51.59, 67.65)	581649.08 (517710.75, 657998.18)	50.90 (45.37, 57.00)	−0.4887 (−0.5351 to −0.4423)
Deaths	High-middle SDI	887525.12 (831446.74, 925960.49)	112.05 (103.37, 116.96)	1151654.57 (1025972.14, 1263429.35)	59.75 (52.99, 65.45)	−2.3994 (−2.6046 to −2.1937)
Deaths	High SDI	595527.75 (535846.62, 623760.21)	53.85 (48.15, 56.57)	507950.08 (426776.86, 553062.11)	19.42 (16.54, 21.03)	−3.5762 (−3.7199 to −3.4323)
Deaths	Western Europe	380194.78 (341008.04, 399352.86)	62.78 (55.96, 66.13)	215655.95 (175954.85, 236145.92)	16.72 (13.85, 18.20)	−4.4291 (−4.5733 to −4.2847)
DALYs	Central Asia	719855.94 (669625.67, 766900.12)	1625.39 (1511.24, 1730.11)	985308.74 (892228.66, 1076484.17)	1356.09 (1234.19, 1474.78)	−1.0371 (−1.3084 to −0.765)
DALYs	High-income Asia Pacific	1927882.90 (1766027.72, 2055873.05)	1047.13 (954.83, 1117.76)	1862336.85 (1582664.05, 2083583.02)	335.36 (287.81, 378.86)	−3.9852 (−4.1441 to −3.8261)
DALYs	Southeast Asia	3004177.44 (2687919.03, 3310660.39)	1355.44 (1203.59, 1499.12)	7318327.82 (6238954.36, 8317299.81)	1266.45 (1088.13, 1430.09)	−0.1711 (−0.2869 to −0.0552)
DALYs	Central Latin America	487685.40 (464997.73, 508604.35)	642.81 (610.09, 669.72)	804658.46 (727628.10, 889436.06)	336.61 (304.38, 372.04)	−2.2859 (−2.4647 to −2.1068)
DALYs	Central Europe	3203847.55 (3074576.63, 3318844.01)	2300.32 (2201.64, 2386.78)	2593397.56 (2385308.67, 2776479.61)	1101.60 (1014.18, 1180.50)	−2.6988 (−2.8364 to −2.5611)
DALYs	Western Sub-Saharan Africa	1138282.35 (925815.74, 1442064.46)	1420.18 (1161.28, 1810.07)	2185272.02 (1840339.51, 2608225.49)	1256.23 (1075.86, 1478.49)	−0.3911 (−0.4942 to −0.2878)
DALYs	High-income North America	2017603.15 (1818785.70, 2187490.40)	554.83 (500.04, 602.52)	2384648.46 (2094174.14, 2640685.46)	352.61 (309.35, 392.65)	−1.9351 (−2.1824 to −1.6872)
DALYs	Southern Sub-Saharan Africa	224900.27 (194622.12, 249787.27)	917.26 (782.58, 1023.35)	523152.90 (480110.08, 569334.03)	1073.71 (979.00, 1165.87)	0.6378 (0.1998 to 1.0776)
DALYs	Tropical Latin America	1095135.72 (1036359.17, 1137132.03)	1368.14 (1271.03, 1425.63)	1292888.02 (1180665.69, 1372019.04)	520.41 (473.32, 553.10)	−3.0494 (−3.1835 to −2.9151)
DALYs	Eastern Sub-Saharan Africa	616224.96 (510230.33, 758430.06)	981.75 (824.06, 1188.93)	1231675.36 (1050098.82, 1440686.19)	873.50 (749.02, 1018.61)	−0.4716 (−0.508 to −0.4353)
DALYs	Central Sub-Saharan Africa	225423.72 (175336.91, 276839.14)	1219.92 (970.28, 1523.56)	447160.61 (347101.51, 579987.09)	1076.29 (830.85, 1416.65)	−0.5402 (−0.5906 to −0.4898)
DALYs	Oceania	22713.59 (18479.82, 28041.15)	937.35 (770.88, 1171.08)	48691.47 (40674.80, 59299.05)	789.38 (663.20, 979.96)	−0.6548 (−0.7132 to −0.5965)
DALYs	Australasia	162897.72 (149973.84, 174032.12)	714.14 (655.92, 763.60)	149125.40 (129769.00, 166038.58)	249.45 (216.51, 278.22)	−3.6136 (−3.7528 to −3.4742)
DALYs	Middle SDI	10950853.74 (9944439.57, 12298162.24)	1221.85 (1103.17, 1364.82)	23896856.70 (21522553.07, 26137544.52)	960.71 (863.98, 1047.82)	−0.8265 (−0.8951 to −0.7578)
DALYs	Andean Latin America	113706.92 (101757.79, 126128.55)	574.17 (516.58, 631.29)	183295.86 (154621.08, 214987.25)	320.11 (270.13, 375.28)	−2.112 (−2.3209 to −1.9028)
DALYs	Caribbean	224864.30 (209623.55, 241939.43)	904.27 (839.81, 968.96)	353682.29 (313628.52, 399100.66)	656.53 (582.42, 741.98)	−0.9744 (−1.0474 to −0.9013)
DALYs	Eastern Europe	7422389.72 (7152141.57, 7643097.84)	2825.98 (2711.24, 2913.40)	5713718.32 (5294961.39, 6142848.26)	1601.20 (1483.51, 1723.12)	−2.6127 (−3.0786 to −2.1445)
DALYs	South Asia	4009489.39 (3328354.16, 5033258.32)	809.31 (676.00, 1013.15)	9193296.62 (8004944.14, 11543774.97)	690.14 (604.04, 851.21)	−0.6699 (−0.7651 to −0.5745)
DALYs	North Africa and Middle East	3002344.39 (2668214.48, 3383384.49)	1940.20 (1715.24, 2180.55)	5405417.35 (4711954.36, 6041794.37)	1329.39 (1165.64, 1483.03)	−1.2331 (−1.2672 to −1.1989)
DALYs	Southern Latin America	424811.29 (394539.83, 451983.71)	974.15 (900.63, 1035.83)	352924.76 (323073.76, 382286.21)	391.27 (357.82, 424.24)	−2.7157 (−2.8445 to −2.5867)
DALYs	Low SDI	2126088.53 (1791117.37, 2645820.05)	1074.89 (910.35, 1345.18)	4059454.62 (3492447.47, 4963611.54)	914.28 (789.76, 1116.62)	−0.5826 (−0.6341 to −0.531)
DALYs	East Asia	10279514.18 (8853619.48, 12005870.44)	1382.70 (1191.08, 1606.73)	24021156.05 (20420316.01, 27562228.89)	1165.93 (998.23, 1336.56)	−0.5342 (−0.7218 to −0.3462)
DALYs	Low-middle SDI	5912050.09 (5266484.05, 6751650.20)	1094.89 (975.43, 1254.57)	12300861.93 (11013179.01, 13998937.35)	942.27 (846.11, 1065.86)	−0.5175 (−0.5549 to −0.4801)
DALYs	High-middle SDI	16876354.80 (15868635.14, 17770724.41)	1885.84 (1768.07, 1980.92)	21054343.33 (19044777.90, 23026603.00)	1076.54 (973.38, 1176.25)	−2.1926 (−2.4 to −1.9847)
DALYs	High SDI	10239251.69 (9461539.39, 10819068.07)	916.44 (845.06, 969.25)	8975176.19 (7913834.94, 9812024.08)	395.56 (352.17, 434.79)	−2.9819 (−3.1185 to −2.8452)
DALYs	Western Europe	5852489.36 (5397741.83, 6160459.15)	963.97 (888.14, 1017.38)	3307776.97 (2903214.36, 3626266.13)	297.71 (262.76, 327.93)	−3.9472 (−4.1076 to −3.7864)

ASR, age-standardized rate (per 100,000 person-years); EAPC, estimated annual percentage change; UI, uncertainty interval; CI, confidence interval; DALYs, disability-adjusted life years; SDI, sociodemographic index; GBD, Global Burden of Disease.

Source: Global Burden of Disease Study 2021.

EAPC indicates the average annual percentage change in ASR from 1990 to 2021.

#### 3.2.2 High-burden geographic regions

Among the 21 GBD regions, Southern Sub-Saharan Africa recorded the highest ASPR (1121.97 per 100,000) in 2021, while Eastern Europe bore the heaviest burden in terms of ASIR (142.57 per 100,000), ASDR (90.99 per 100,000), and DALY rate (1601.20 per 100,000). Comprehensive regional estimates are provided in Table 2.

### 3.3 National-level analysis

#### 3.3.1 Country rankings for key metrics in 2021

**Figure 2:**
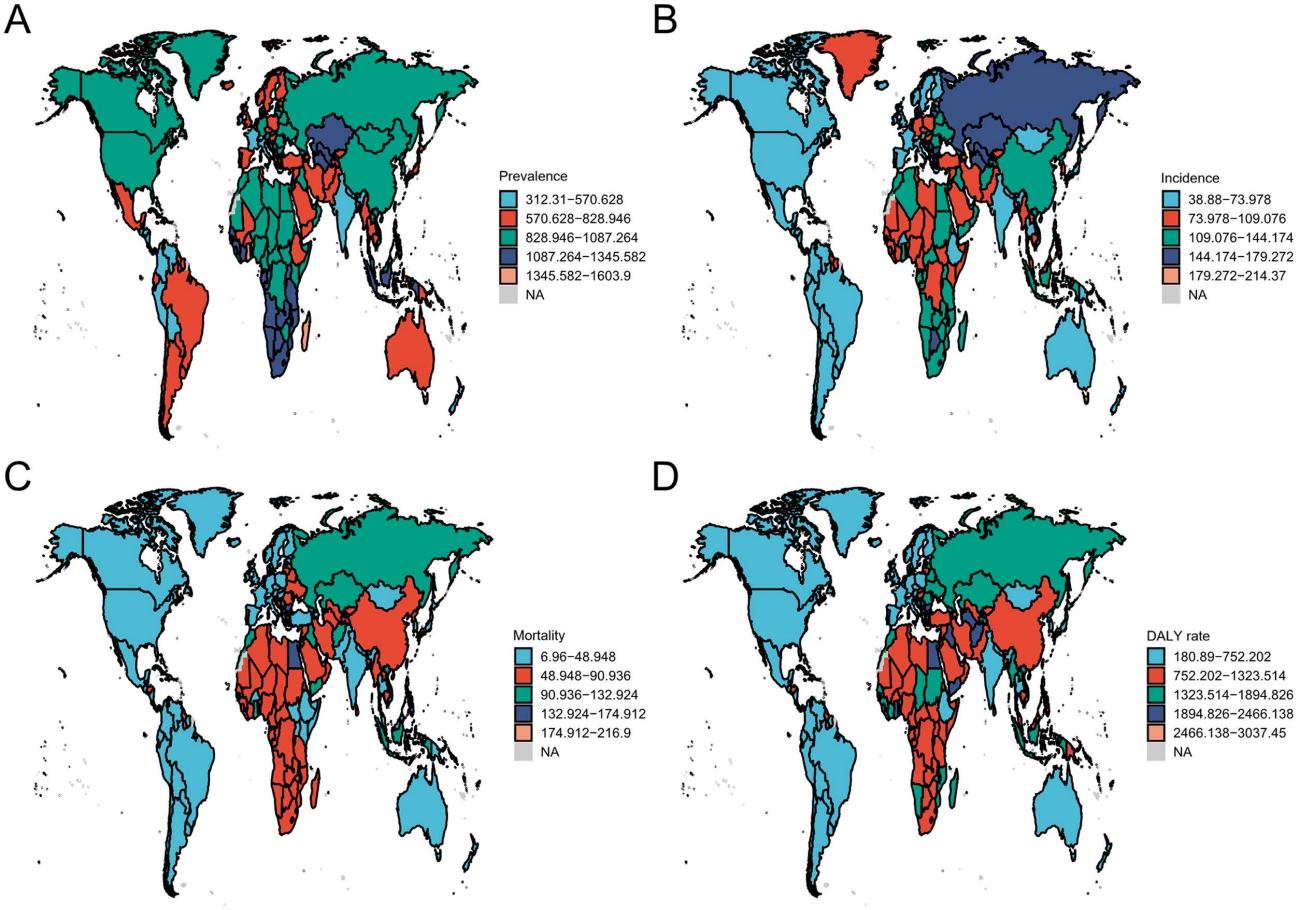
Burden of ischemic stroke across 204 countries and territories. **(A)** Prevalence. **(B)** Incidence. **(C)** Mortality. **(D)** DALY rate.

In 2021, significant variations in ASPR were evident across 204 countries and territories globally. Ghana had the highest ASPR (1603.89 per 100,000), while Cyprus recorded the lowest (312.32 per 100,000). For incidence, North Macedonia ranked first (214.36 per 100,000) and Malta last (38.89 per 100,000). The highest mortality rate was also observed in North Macedonia (216.89 per 100,000), and the lowest in Singapore (6.97 per 100,000). DALY rates followed a similar pattern, with the highest in North Macedonia (3,037.44 per 100,000) and the lowest in Puerto Rico (180.90 per 100,000). Complete country-specific data are presented in Figure 2.

#### 3.3.2 Magnitude of change at the national level (1990–2021)

**Figure 3:**
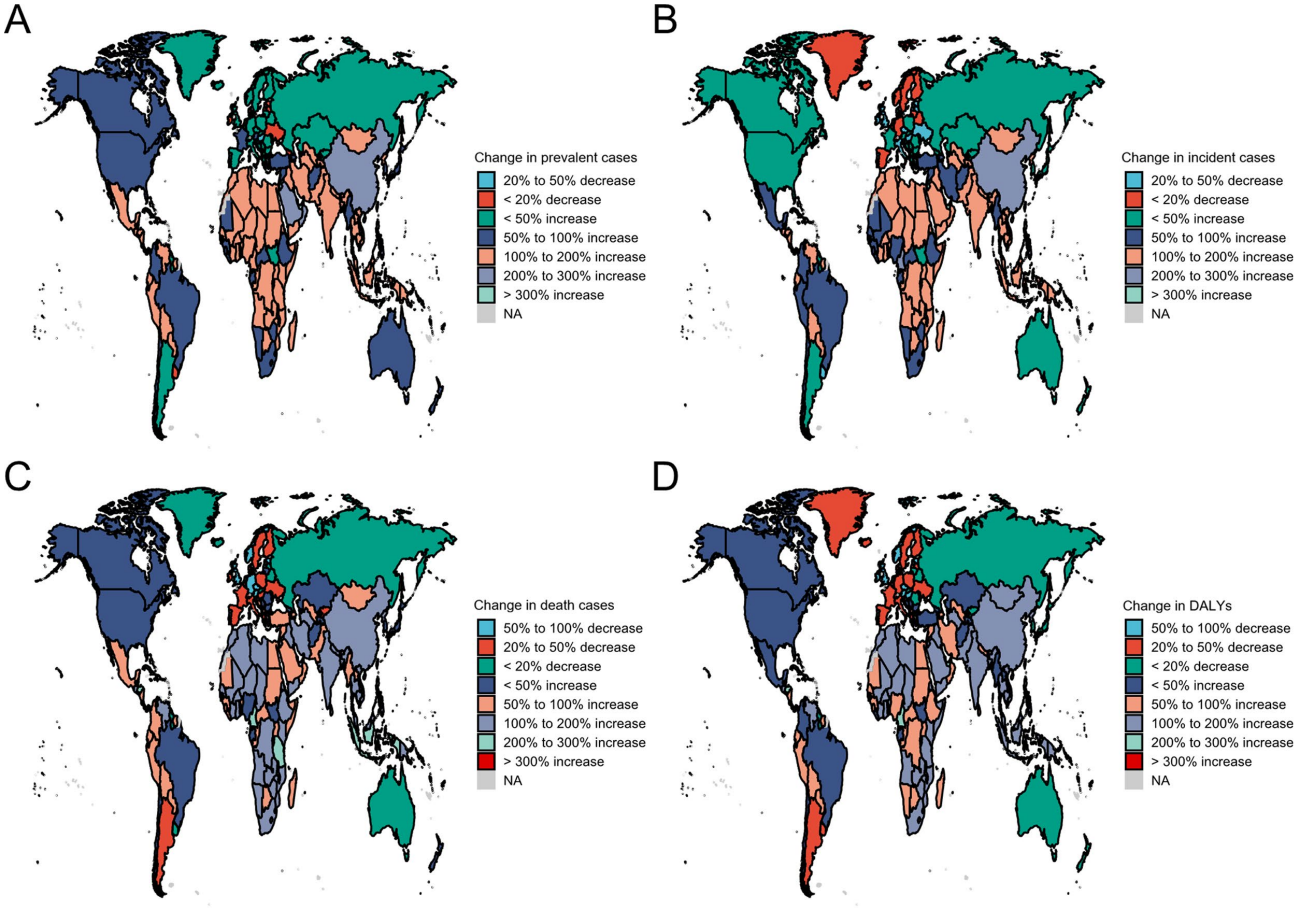
Changes in the burden of ischemic stroke across 204 countries and territories. **(A)** Change in prevalent cases. **(B)** Change in incident cases. **(C)** Change in death cases. **(D)** Change in DALYs.

Between 1990 and 2021, substantial differences in disease burden trends were observed at the national level. The United Arab Emirates experienced the largest relative increase in prevalent cases (+677.18%), whereas Portugal showed the greatest decline (−33.11%). Incident cases rose fastest in the United Arab Emirates (+652.67%) and fell most sharply in Estonia (−44.19%). For deaths, Djibouti recorded the largest increase (+373.46%) and Estonia the largest decrease (−67.23%). DALYs followed the same pattern, with the highest increase in Djibouti (+349.18%) and the steepest decline in Estonia (−68.97%). These national variations are illustrated in Figure 3.

### 3.4 Age and gender-specific patterns

#### 3.4.1 Age at peak incidence by gender and SDI

**Figure 4:**
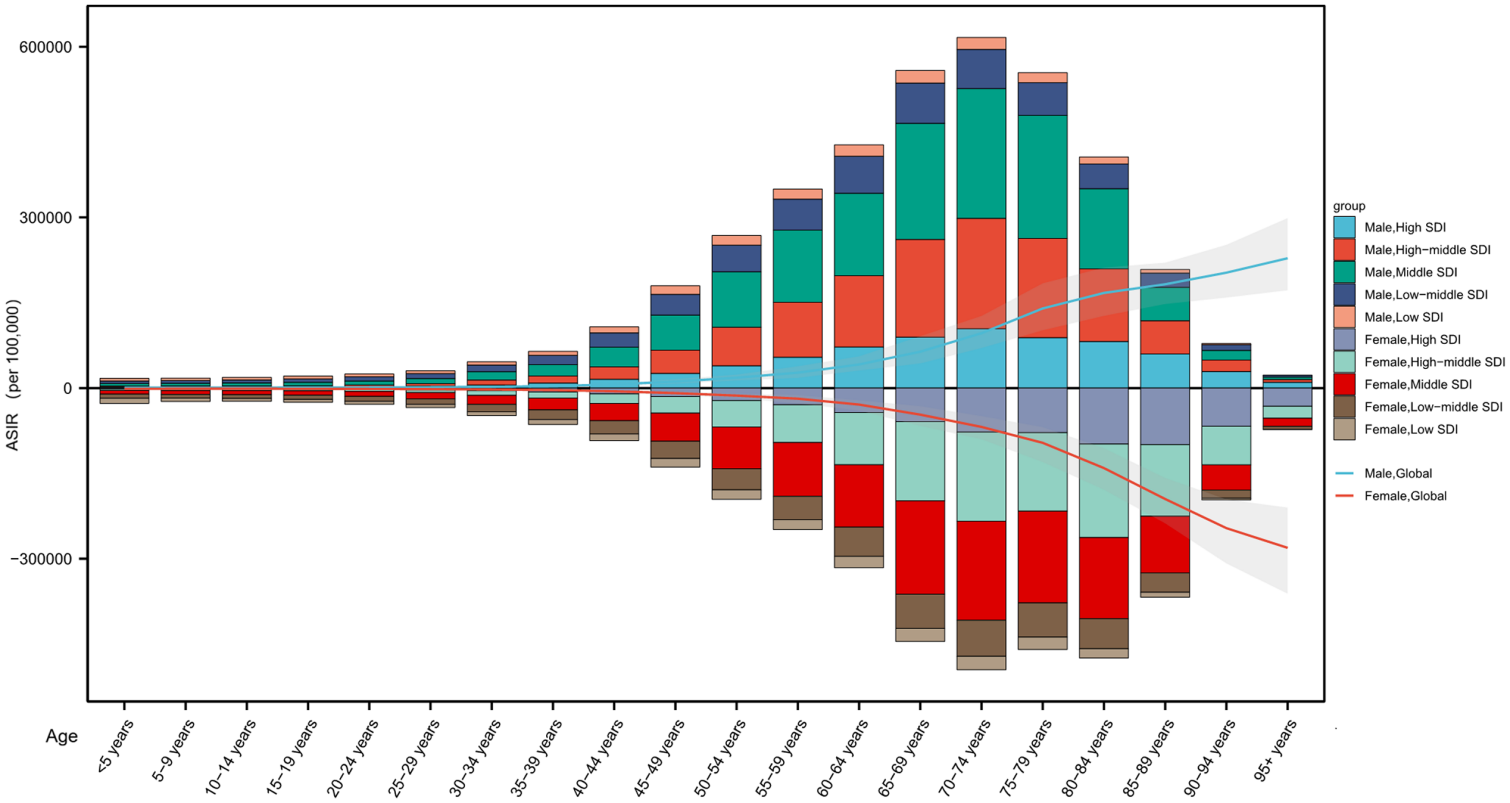
Age-specific numbers and age-standardized incidence rates (ASIRs) by sociodemographic index (SDI) region in 2021.

Analysis of incident cases across genders and SDI levels revealed that the peak age group for male incidence predominantly ranged from 65 to 79 years. Specifically, in high SDI regions, males aged 70–74 years had the highest number of incident cases at 104240.15 (77720.60–134380.30), followed by the 65–69 years group. In high-middle SDI regions, the 70–74 years group peaked at 194079.33 (142185.86–254827.17), with the 75–79 years group next highest. Middle SDI males aged 70–74 years had 228237.68 (165123.05–299030.38) incident cases, slightly exceeding the 75–79 years group. In low-middle SDI regions, the highest incidence was among 65–69 years at 70892.81 (48485.10–100012.98), followed by 70–74 years. Low SDI males peaked at 65–69 years with 22094.85 (15099.99–31169.64), closely followed by 70–74 years (Figure 4).

For females, the peak incidence age was generally higher. In high SDI regions, females aged 85–89 years had the highest incident cases at 99296.07 (81902.84–119652.63), followed by 80–84 years. High-middle SDI females peaked at 80–84 years with 164520.70 (123841.77–209141.89), then 70–74 years. Middle SDI females aged 70–74 years had the highest incidence at 173824.26 (122259.51–232674.80), followed by 65–69 years. Both low-middle and low SDI females showed peak incidence at 70–74 years, with 63630.58 (45743.97–83979.49) and 23629.13 (17325.69–31319.49), respectively, followed by 75–79 and 65–69 years groups, respectively.

#### 3.4.2 Temporal shifts in age at peak prevalence

**Figure 5:**
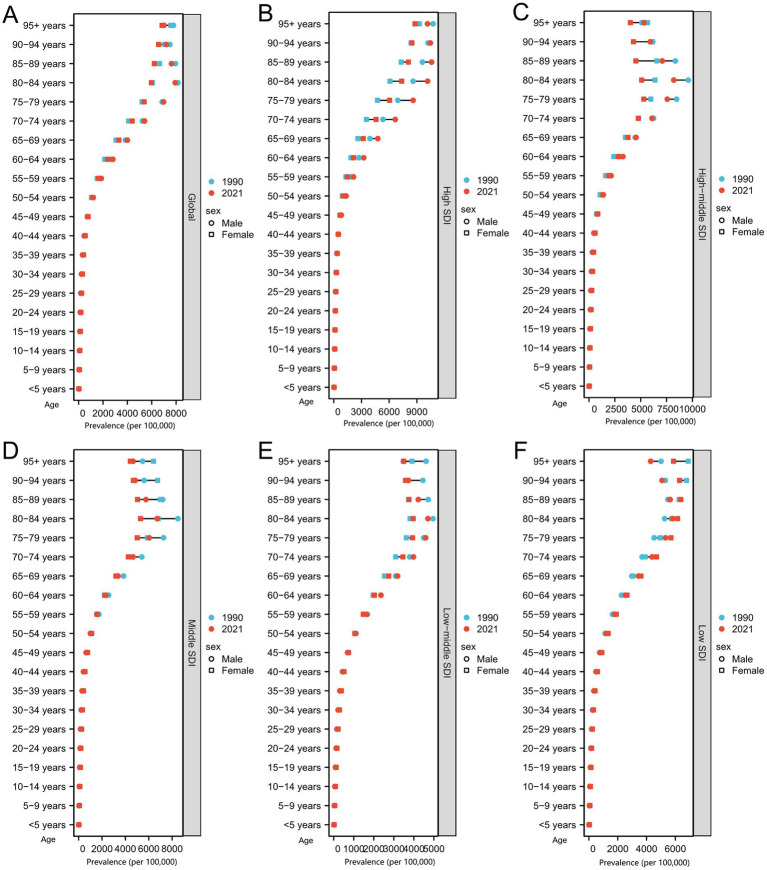
Prevalence by sex, age group, and sociodemographic index (SDI) in 1990 and 2021. **(A)** Global. **(B)** High SDI region. **(C)** High-middle SDI region. **(D)** Middle SDI region. **(E)** Low-middle SDI region. **(F)** Low SDI region.

Ischemic stroke prevalence data indicated that in 1990, the highest male prevalence globally was in the 80–84 years group at 7922.27, slightly increasing to 8151.69 in 2021. Females had the highest prevalence in the ≥95 years group, rising from 6850.47 in 1990 to 7564.08 in 2021. In high SDI regions, male peak prevalence shifted from 85–89 years (10591.72 in 1990) to ≥95 years (10754.73 in 2021). Female prevalence remained highest in the ≥95 years group, increasing from 8803.72 to 9260.69. In high-middle SDI regions, male peak prevalence was at 80–84 years, increasing from 8192.16 to 9595.91, while female peak prevalence shifted from 75–79 to 85–89 years. Middle SDI males also showed an increase in 80–84 years prevalence from 6725.16 to 8498.37, with females’ peak prevalence moving from 80–84 to 85–89 years. Low-middle SDI males had a slight increase at 80–84 years, and female peak prevalence shifted from 80–84 to ≥95 years. In low SDI regions, male peak prevalence shifted from 80–84 to 85–89 years, and female peak prevalence extended from 85–89 to ≥95 years (Figures 5A–F).

There are clear differences in peak incidence ages by gender and SDI level, with males generally peaking earlier than females, and female peak incidence tending toward older age groups. These patterns may be influenced by biological sex differences, lifespan disparities, and regional healthcare resource distribution. Additionally, a temporal trend toward older peak prevalence ages in some regions may reflect population aging and improvements in healthcare.

### 3.5 Future projections of global burden

**Figure 6:**
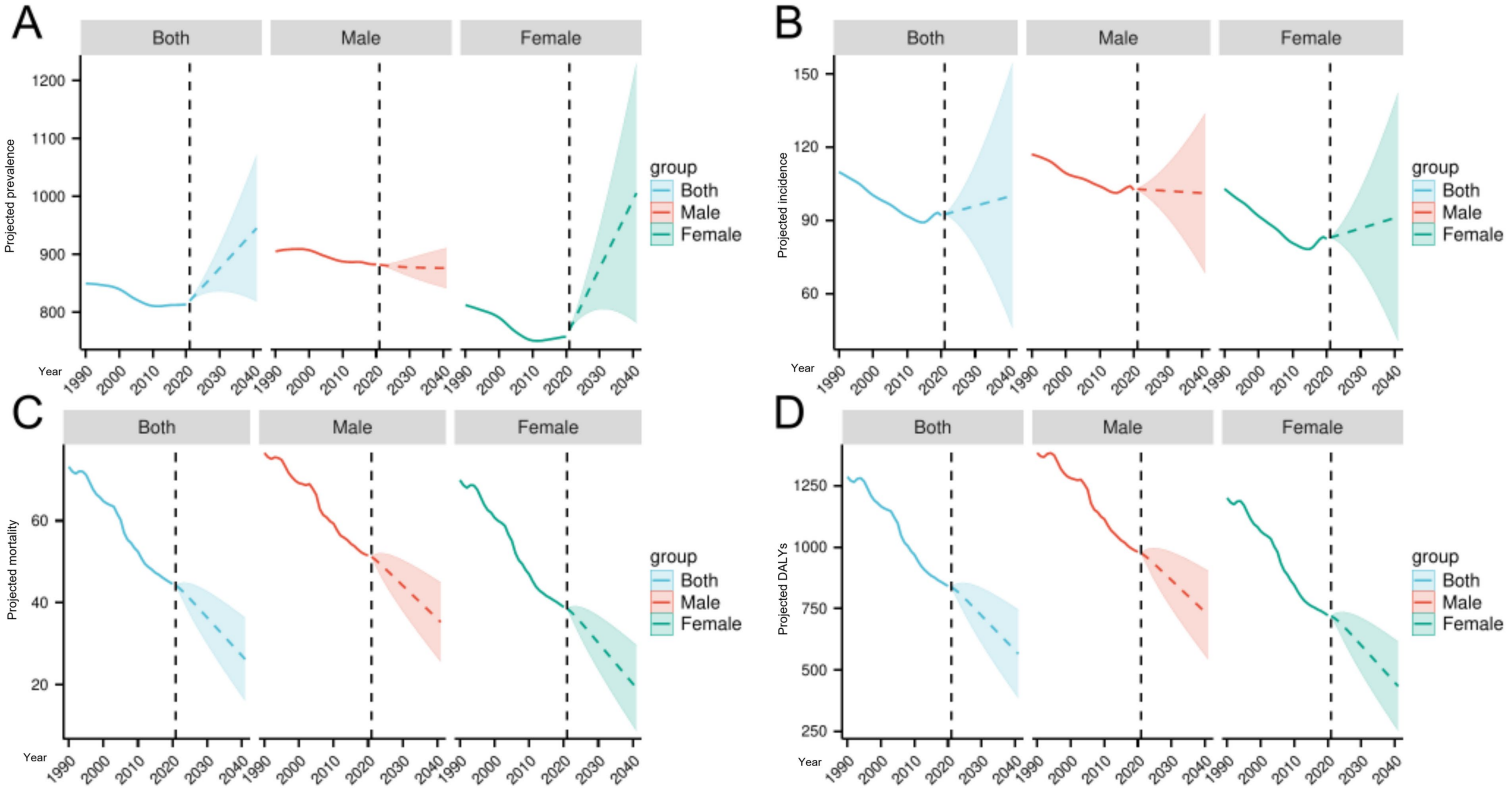
Future projections of the global burden of ischemic stroke. **(A)** Projected prevalence. **(B)** Projected incidence. **(C)** Projected mortality. **(D)** Projected DALYs.

From 2021 to 2041, the global burden of ischemic stroke is projected to exhibit complex changes. Overall, the global ASPR is expected to rise from approximately 819.47 per 100,000 in 2021 to 944.85 per 100,000 in 2041, an increase of about 15.3%. Male ASPR is predicted to slightly decline from around 881.94 to 876.09, whereas female ASPR is projected to increase markedly from about 769.40 to 1005.77 (Figure 6A). ASIR globally is forecasted to increase from 92.39 to 100.12 per 100,000 (an 8.36% increase), with male ASIR slightly decreasing from 102.77 to 101.09 and female ASIR rising from 82.85 to 91.37 (Figure 6B). Both ASDR and DALYs are projected to decline substantially; ASDR is expected to decrease by approximately 40.68%, from 44.18 to 26.21 per 100,000, with male ASDR falling from 51.16 to 35.27 and female ASDR from 38.54 to 19.23 (Figure 6C). DALYs are also predicted to reduce by about 32.46%, from 837.36 to 565.52 per 100,000, with male DALYs decreasing from 975.30 to 723.23 and female DALYs from 719.52 to 434.08 (Figure 6D).

These trends suggest that, although prevalence and incidence rates are expected to increase among females, overall mortality and disease burden are projected to decline, particularly among males. This may reflect advancements in medical technology, wider adoption of preventive measures, and improved disease management. The observed sex differences may be attributable to biological factors, lifestyle changes, and unequal healthcare resource distribution. Future research should prioritize understanding risk factor dynamics in females and developing targeted interventions to effectively reduce ischemic stroke burden.

## 4 Discussion

### 4.1 Principal findings and global context

This study provides a comprehensive analysis of the global burden of ischemic stroke from 1990 to 2021, revealing significant temporal and geographical patterns. Despite an overall decline in age-standardized prevalence, incidence, mortality, and DALYs of ischemic stroke globally, substantial disparities exist across regions and sociodemographic index (SDI) levels. High SDI regions demonstrated the most pronounced decreases in mortality and DALYs, while middle SDI regions showed increasing trends in prevalence and incidence. This complex pattern reflects the uneven distribution of stroke prevention and management resources worldwide and highlights the necessity of region-specific control strategies to effectively address the global challenge of ischemic stroke.

### 4.2 Interpretation of regional disparities

Our study revealed substantial regional disparities in ischemic stroke burden globally. Eastern Europe, East Asia, and Southern sub-Saharan Africa demonstrated notably higher disease burdens, while Western Europe and high-income North America exhibited relatively lower rates. These disparities are driven by multiple factors, including unequal healthcare resource distribution, varying implementation of preventive strategies, lifestyle differences, and demographic transitions. Specifically, healthcare access inequalities significantly influence stroke outcomes, as evidenced by studies demonstrating the correlation between health expenditure, human development indices, and stroke rates (18). The urban–rural divide observed in our findings mirrors patterns documented in Japan, where despite overall declining stroke incidence over five decades, rural communities consistently showed 1.7–2.2 times higher rates than urban areas (19). The rising incidence in middle-SDI regions (EAPC = 0.12) reflects a classic epidemiological double burden. These regions are undergoing rapid urbanization and lifestyle transitions—increasing prevalence of hypertension, obesity, and diabetes—while healthcare systems remain oriented toward acute care rather than primary prevention. Unlike high-SDI regions, which have built infrastructure for population-wide risk factor control over decades, middle-SDI regions face a convergence of emerging risk factors and unprepared health systems. This creates a critical window: without immediate investment in hypertension screening and treatment, the rising incidence will translate into an accelerating burden of chronic stroke disability. This phenomenon creates a “double burden,” where these regions face increasing exposure to modern risk factors such as hypertension and obesity while still lacking adequate preventive infrastructure. Additionally, regional variations may be influenced by the prevalence of specific risk factors, as demonstrated by a Chinese study identifying chronic hepatitis B infection as a significant risk factor for intracerebral hemorrhage in certain areas (20). Collectively, these findings underscore the need for geographically tailored intervention strategies that address both socioeconomic determinants of health and region-specific risk factors to effectively reduce the global burden of ischemic stroke.

### 4.3 Gender and age disparities: underlying mechanisms

We further observed significant gender and age-related differences in the burden of ischemic stroke. Men generally experienced earlier peak incidence compared to women, with most SDI regions showing higher age-standardized incidence rates in men. This gender disparity may be attributed to several underlying factors. Biological mechanisms, particularly hormonal differences, likely play a substantial role, as estrogen has been shown to provide neuroprotective effects in women prior to menopause (21). The loss of this protection after menopause may increase women’s susceptibility to ischemic injury and adversely influence post-stroke recovery and long-term outcomes (22). Behavioral factors are also important, with higher prevalence of smoking and alcohol consumption among men contributing to their elevated stroke risk (23).

In addition to these traditional risk factors, female-specific reproductive factors may contribute to the long-term stroke burden in women. Conditions such as preeclampsia, gestational diabetes, and hypertensive disorders of pregnancy are established risk factors for future cerebrovascular disease (24–26). Furthermore, the use of oral contraceptives, particularly among younger women, has been associated with a modest increase in stroke risk, especially in those with concomitant risk factors such as smoking or migraine (27). Migraine with aura—a condition with higher prevalence in women—has been associated with increased stroke risk (28). These exposures may have cumulative effects that manifest only decades later, contributing to the rising prevalence observed in older women.

Beyond risk factor profiles, differences in acute treatment response and post-stroke care may influence long-term survival and thus prevalence. Studies have suggested that women may present with more severe strokes, experience delays in hospital arrival compared to men (29). Even when treated, women may have poorer functional outcomes and higher disability rates, which could contribute to the growing pool of prevalent cases as they survive the acute event but live with long-term sequelae (30).

The age-related patterns observed in our study revealed an interesting shift in the peak prevalence age for women toward older age groups over time, particularly evident in high SDI regions. This trend likely reflects both increased female longevity and the loss of hormonal protection after menopause (22). Age-period-cohort analyses further support these observations, showing distinctive patterns between genders that evolve with age (31). These results are consistent with previous research indicating that stroke etiologies differ significantly between genders, with women more frequently experiencing cardioembolic stroke and men more commonly suffering from large and small vessel disease (32).

Taken together, the projected surge in female stroke prevalence—from 769.40 to 1005.77 per 100,000 by 2041—reflects a convergence of biological vulnerability (post-menopausal hormonal loss), cumulative reproductive exposures (preeclampsia, oral contraceptives), and health system factors (disparities in acute care). This multifactorial etiology suggests that single-factor interventions will be insufficient; instead, gender-sensitive strategies must address the entire female life course, from reproductive health to post-menopausal risk factor management and equitable acute stroke care.

### 4.4 The complex relationship with socioeconomic development

The relationship between sociodemographic index (SDI) levels and ischemic stroke burden demonstrates a complex, non-linear pattern across global regions. High SDI regions have experienced the most significant reductions in mortality and disability-adjusted life years (DALYs), with death rates declining by an estimated annual percentage change (EAPC) of −3.58 and DALY rates decreasing by an EAPC of −2.98. This substantial improvement is likely attributable to well-established healthcare systems that provide effective secondary prevention measures and acute stroke management (33). These regions benefit from advanced medical technologies, specialized stroke units, and comprehensive rehabilitation services that significantly improve survival rates and functional outcomes following stroke events (34).

In contrast, middle SDI regions present a concerning pattern of increasing prevalence (EAPC: 0.27) and incidence rates (EAPC: 0.12), potentially reflecting their position in the epidemiological transition. On one hand, these regions are undergoing rapid urbanization and lifestyle changes—rising caloric intake, declining physical activity, and increasing prevalence of hypertension, obesity, and type 2 diabetes (35). On the other hand, their healthcare systems remain largely oriented toward acute care rather than population-level prevention. This creates a critical gap: risk factors are shifting toward those of high-income countries, but the public health infrastructure to manage these risks has not yet caught up (36). The healthcare infrastructure in these regions may be inadequately prepared to address the growing stroke burden, particularly in terms of specialized stroke care and rehabilitation services.

Low SDI regions currently report relatively lower ischemic stroke burden compared to middle and high SDI regions, but this may represent underdiagnosis rather than true lower prevalence. As these regions undergo demographic and epidemiological transitions, their stroke burden is likely to increase substantially in coming decades due to population aging, increasing life expectancy, and adoption of high-risk lifestyles (37). The observed “SDI gradient” in stroke burden suggests that preventive strategies must be tailored to regions’ specific developmental stages, with high SDI regions focusing on maintaining progress through technological innovation, middle SDI regions urgently needing to strengthen healthcare capacity while implementing primary prevention, and low SDI regions requiring early investment in surveillance and prevention infrastructure to mitigate future burden increases.

### 4.5 The paradox of improving care and rising prevalence

A central finding of this study—declining mortality alongside rising prevalence—reflects a fundamental tension in contemporary stroke epidemiology that is often obscured when focusing solely on age-standardized rates. On one hand, improvements in acute stroke care—wider availability of thrombolysis, mechanical thrombectomy, and specialized stroke units—have substantially reduced case fatality rates, particularly in high- and high-middle SDI regions. More patients survive the acute event, which is a clear success. On the other hand, many survivors live with residual disability, joining the growing pool of prevalent cases. This creates what might be termed a “treatment success paradox”: the very interventions that reduce mortality also increase prevalence, as more individuals live longer with post-stroke sequelae (38). Compounding this dynamic is population aging. Even as age-standardized rates decline in many regions, the absolute number of stroke cases continues to rise because the population at risk—older adults—is growing. In high-SDI regions, where age-standardized mortality has fallen by more than 60% since 1990, the absolute number of stroke survivors has increased by nearly 50% over the same period (1). This demographic reality means that health systems must plan not only for acute stroke treatment but also for long-term rehabilitation, chronic disease management, and caregiver support. The projected rise in prevalence through 2041 suggests that the burden of post-stroke disability will continue to grow even as medical care improves, requiring a shift in resource allocation from acute-only models toward integrated stroke care that spans prevention, acute treatment, and long-term support.

### 4.6 Implications of future projections

Looking ahead, our ARIMA model-based predictions suggest that global ischemic stroke burden will follow a complex trajectory over the next two decades. Age-standardized prevalence rates are projected to increase by approximately 15.3% from 2021 to 2041, with a particularly notable rise in the female population (from 769.40 to 1005.77 per 100,000). Similarly, age-standardized incidence rates are expected to increase by 8.36% overall, again with a more pronounced increase among females. In contrast, both mortality rates and DALYs are predicted to decrease substantially (by 40.68 and 32.46%, respectively). The growing gender disparity in our projections, with women experiencing steeper increases in prevalence and incidence while simultaneously achieving greater reductions in mortality, warrants particular attention. This pattern suggests that while acute care improvements have benefited both sexes, the underlying risk burden—particularly from female-specific exposures and post-menopausal hormonal changes—may be driving a widening gap in disease prevalence. These findings underscore the need for gender-specific prevention and management strategies that address the distinct risk profiles of women across the life course.

Similar trends have been observed in other stroke prediction studies, which have also noted the importance of demographic shifts and medical advancements in shaping future stroke epidemiology (39, 40). However, our study is the first to provide sex-specific forecasts to 2041 at the global level, offering a more granular basis for health system planning. These forecasts emphasize that while acute stroke management has improved survival outcomes, greater emphasis on primary prevention is needed to address the anticipated rise in new cases, particularly among women. The predictive approach used aligns with established methodologies, though it should be noted that such models cannot fully account for potential future changes in risk factor prevalence or treatment breakthroughs.

### 4.7 Public health implications

Our findings have significant public health implications by providing a comprehensive and updated panorama of the global burden of ischemic stroke. This information enables governments and international organizations to optimize healthcare resource allocation based on evidence-driven priorities. The regional disparities we identified highlight the necessity for tailored prevention strategies, such as intensifying risk factor management in high-burden regions and strengthening health education and primary prevention in middle SDI areas where incidence rates are rising. Furthermore, our observations regarding gender and age differences provide valuable insights for precision medicine approaches and individualized preventive interventions, particularly recognizing the shifting patterns of stroke burden toward older age groups, especially among women. Additionally, our future trend predictions offer a scientific foundation for long-term health policy planning, allowing countries to proactively address anticipated challenges, including the projected increase in prevalence among women despite overall mortality reductions. These findings collectively emphasize the importance of context-specific approaches to stroke prevention and management that account for sociodemographic, regional, and gender-based variations in disease burden.

### 4.8 Limitations

Several limitations exist in the present study. First, as research based on the GBD database, its accuracy depends on the quality and coverage of the original data, with potential missing or quality issues in data from low SDI regions. This underdiagnosis in low-resource settings likely reflects more than just data gaps—it probably represents substantial underestimation of the true burden. In many low-income countries, limited access to healthcare facilities, scarcity of neuroimaging, and high rates of out-of-hospital mortality mean that a large proportion of stroke cases are never formally diagnosed or recorded in health statistics. This under-ascertainment has critical implications for both current estimates and future projections. Current GBD estimates for low SDI regions are almost certainly underestimates of the true burden, as they rely on data that systematically miss cases occurring outside formal healthcare systems. More importantly, as healthcare infrastructure and diagnostic capacity improve in these regions—with expanded access to CT imaging, stroke units, and trained personnel—we anticipate a substantial artificial increase in reported stroke cases over the coming decades. This could potentially outpace current model predictions, which are calibrated on historically under-ascertained data. Investment in population-based stroke surveillance and vital registration systems is urgently needed to accurately track this epidemiological transition and prepare healthcare systems for the true scale of the coming burden. Second, while our prediction models considered historical trends, they cannot fully anticipate the impacts of future public health interventions, medical technological breakthroughs, or significant social changes. Third, this study did not differentiate between various subtypes of ischemic stroke, which may have different risk factors and changing patterns. Finally, as an ecological study, our findings cannot directly infer causality at the individual level. Future research should incorporate more primary data and prospective studies to further validate the findings of this study.

### 4.9 Future research directions

Based on the findings and limitations of our study, future research should focus on several key directions to advance our understanding and management of ischemic stroke burden. First, there is a critical need to explore the specific mechanisms underlying regional disparities in ischemic stroke burden, particularly investigating the relative contributions of socioeconomic factors versus healthcare system characteristics. Second, more detailed classification studies of different ischemic stroke subtypes are essential to develop more precise prevention strategies tailored to specific pathophysiological mechanisms. Third, dedicated research on gender disparities is warranted, with special attention to female-specific risk factors and protective factors that may explain the observed epidemiological differences. Fourth, cost-effectiveness evaluations of various prevention and intervention strategies should be prioritized to provide evidence-based best practice recommendations for regions with limited resources. Finally, integrating genomic data with environmental factors could lead to the development of individualized prediction models for ischemic stroke risk across different populations, advancing the field toward more personalized prevention approaches. These research directions would address current knowledge gaps and potentially lead to more effective strategies for reducing the global burden of ischemic stroke.

## 5 Conclusion

This study provides a comprehensive evaluation of the global burden of ischemic stroke from 1990 to 2021, revealing that while age-standardized prevalence, incidence, mortality, and DALY rates have generally declined, significant disparities persist across regions, sociodemographic index (SDI) levels, genders, and age groups. High SDI regions have experienced the most pronounced reductions in mortality and disability, whereas middle SDI regions show rising prevalence and incidence, reflecting uneven distribution of healthcare resources and emerging epidemiological transitions. Projections for the next two decades indicate a continued increase in prevalence and incidence globally—especially among women—contrasted with substantial declines in mortality and DALYs. These nuanced trends highlight the complex interplay between demographic shifts, healthcare advances, and socioeconomic factors influencing ischemic stroke burden worldwide.

Based on these findings, tailored public health strategies are imperative. High SDI countries should consolidate gains by enhancing secondary prevention and rehabilitation services, while middle SDI regions need to prioritize primary prevention to curb the accelerating risk factors associated with urbanization and lifestyle changes. Low SDI regions require integrated approaches addressing both communicable and non-communicable disease burdens, alongside strengthening healthcare infrastructure. Importantly, gender-specific interventions addressing the distinct risk profiles and disease trajectories in women are essential. Multisectoral collaboration and international cooperation will be critical to optimize resource allocation and implement context-specific prevention and treatment programs. Such targeted efforts hold promise for mitigating ischemic stroke’s global impact, alleviating its socioeconomic burden, and advancing equitable health outcomes across diverse populations.

## Data Availability

The original contributions presented in the study are included in the article/supplementary material, further inquiries can be directed to the corresponding authors.

## Footnotes

1
https://ghdx.healthdata.org/
